# Analysis of individual cells identifies cell‐to‐cell variability following induction of cellular senescence

**DOI:** 10.1111/acel.12632

**Published:** 2017-07-11

**Authors:** Christopher D. Wiley, James M. Flynn, Christapher Morrissey, Ronald Lebofsky, Joe Shuga, Xiao Dong, Marc A. Unger, Jan Vijg, Simon Melov, Judith Campisi

**Affiliations:** ^1^ Buck Institute for Research on Aging Novato CA 94945 USA; ^2^ Fluidigm Corporation South San Francisco CA 94080 USA; ^3^ Department of Genetics Albert Einstein College of Medicine Bronx NY 10461 USA; ^4^ Lawrence Berkeley National Laboratory Berkeley CA 94720 USA

**Keywords:** aging, cellular senescence, cytokines, single cell, transcriptomics

## Abstract

Senescent cells play important roles in both physiological and pathological processes, including cancer and aging. In all cases, however, senescent cells comprise only a small fraction of tissues. Senescent phenotypes have been studied largely in relatively homogeneous populations of cultured cells. *In vivo*, senescent cells are generally identified by a small number of markers, but whether and how these markers vary among individual cells is unknown. We therefore utilized a combination of single‐cell isolation and a nanofluidic PCR platform to determine the contributions of individual cells to the overall gene expression profile of senescent human fibroblast populations. Individual senescent cells were surprisingly heterogeneous in their gene expression signatures. This cell‐to‐cell variability resulted in a loss of correlation among the expression of several senescence‐associated genes. Many genes encoding senescence‐associated secretory phenotype (SASP) factors, a major contributor to the effects of senescent cells *in vivo*, showed marked variability with a subset of highly induced genes accounting for the increases observed at the population level. Inflammatory genes in clustered genomic loci showed a greater correlation with senescence compared to nonclustered loci, suggesting that these genes are coregulated by genomic location. Together, these data offer new insights into how genes are regulated in senescent cells and suggest that single markers are inadequate to identify senescent cells *in vivo*.

## Introduction

Cellular senescence is a process by which mitotically competent cells permanently arrest proliferation (growth) in response to a variety of physiological signals and pathological stresses (Munoz‐Espin & Serrano, 2014). Because the growth arrest prevents the propagation of stressed or damaged cells, the senescence response is an important tumor‐suppressive mechanism (Campisi, 2013). Further, because senescent cells accumulate with age, they can cause or contribute to several degenerative diseases of aging (Baker *et al*., 2016). These effects might stem from the fact that senescent cells cannot divide and therefore cannot create new cells to maintain tissue homeostasis. However, as senescent cells generally comprise a minority of cells within even very old tissues (Dimri *et al*., 1995; Herbig *et al*., 2006; Kreiling *et al*., 2011; Waaijer *et al*., 2012; Baker *et al*., 2016), it is more likely that senescent cells drive age‐related disease cells nonautonomously. Indeed, senescent cells secrete a myriad of inflammatory cytokines, chemokines, proteases, and growth factors that can have potent effects on tissue microenvironments (Coppe *et al*., 2008) and thus drive age‐related pathologies by mechanisms that extend beyond the loss of proliferative potential.

Traditional gene expression analyses that compare transcriptional profiles of cell populations are limited because they measure average of gene expression levels across the entire population. For example, two populations of 5000 cells each might show a twofold difference in the mRNA level of a particular gene, but this change could result from every cell expressing twice as much mRNA, or from a single cell expressing 5000 times more of that mRNA. The difference between these possibilities could have enormous phenotypic consequences in the context of a tissue. Single‐cell approaches offer advantages over population studies because they can distinguish between these types of scenarios. Single‐cell analyses also require fewer cells and therefore can be used to interrogate the phenotypes of rare cells, such as senescent cells produced during organismal aging.

Senescent cells display many qualities that make them desirable candidates for single‐cell transcriptional analysis. As noted above, they are typically rare (Dimri *et al*., 1995; Herbig *et al*., 2006; Kreiling *et al*., 2011; Waaijer *et al*., 2012; Baker *et al*., 2016) and also often occur alongside nonsenescent cells. Further, many of the phenotypic differences between senescent cells and their nonsenescent counterparts are due to changes in mRNA levels, including mRNAs encoding many SASP factors (Coppe *et al*., 2008).

To assess the contributions of individual senescent cells to known senescent phenotypes, we conducted quantitative PCR analyses of single quiescent and senescent cells from cultured populations of human fibroblasts. From these analyses, we find that (i) virtually all senescent cells display a gene expression signature that distinguishes them from their quiescent counterparts; (ii) nonetheless, the expression of most genes is more variable in senescent cells compared to quiescent cells; and (iii) there are correlations among genes expressed by senescent cells, including those encoding SASP factors, that localize in genomic clusters. Together, the data demonstrate that senescent phenotypes are more variable than the transcriptional profiles of cell populations previously suggested.

## Results

### Single senescent cells can be identified by gene expression signatures

Although cellular senescence is marked by strong phenotypic and gene expression changes at the population level, less is known about how individual cells contribute to the overall gene expression pattern. We therefore induced senescence in IMR‐90 human fibroblasts using the clastogen bleomycin (Fig. S1A) (Orjalo *et al*., 2009) and analyzed individual senescent cells using a commercially available platform from Fluidigm (C1) or manual isolation (MI). To control for growth status, we cultured nonsenescent cells for 3 days in 0.2% FBS to induce quiescence (Fig. S1A). Senescence was confirmed by senescence‐associated beta‐galactosidase (SA‐Bgal) activity (Dimri *et al*., 1995), markedly reduced EdU incorporation into nuclear DNA (a measure of nuclear DNA replication) (Fig. S1B), and secretion of a major SASP factor, interleukin‐6 (IL‐6) (Fig. S1C).

C1 isolation captured 57 (59%) single senescent cells and 79 (82%) quiescent cells. The different capture efficiencies were like due to the increased size and irregular shape of senescent cells, resulting in several empty wells. In addition, we manually isolated (MI) 163 single senescent and 156 single quiescent cells.

We analyzed the cells for expression (mRNA levels) of 96 genes, including those associated with cell cycle arrest (12 genes), nuclear lamins (four genes), mitochondria and energy production (12 genes), the SASP (46 genes), signal transduction (four genes), and other senescence regulators (11 genes), in addition to seven control genes, the expression of which were expected to be relatively invariant (Data S1). We chose these genes mainly based on prior knowledge of gene expression associated with cellular senescence. Of the 96 genes analyzed, we excluded 25 as either nonamplifying (no or late Ct) or nonspecific (multiple melting curves), resulting in a dataset comprised of 71 gene expression (mRNA) levels in 235 quiescent and 229 senescent cells. These 71 transcripts were detected in at least 30% of the analyzed cells, with many of them being detected in > 60% of the analyzed cells (Fig. S2A). Although several transcripts (e.g., *CXCL2*,* MMP8, IGFBP6*) were detected at a higher percentage in the C1 dataset, most transcripts were detected at higher percentage in the MI dataset (Fig. S2B). By comparison, fewer differences were seen in detection percentages between senescent and quiescent datasets, with a few exceptions (Fig. S2C). For example, for *LMNB1* gene expression—which declines in senescent cells (Freund *et al*., 2012)—there were fewer senescent cells in which the transcript was detected compared to transcript detection in quiescent cells. Conversely, *TNFRS10C* gene expression, which is induced in senescent cells (Coppe *et al*., 2008), was detected in a higher percentage of senescent cells relative to quiescent cells (Fig. S2C). A few highly studied SASP factors, such as IL‐1B, IL‐6, IL‐7, CXCL1‐3, MMP1, MMP10, and CCL13, were also detected at low percentages (Fig. S2A), limiting the types of analyses that could be performed on these factors.

**Figure 1:**
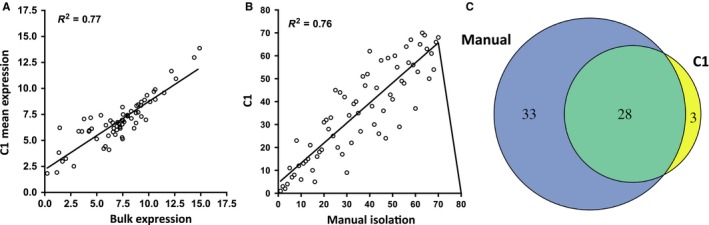
Comparison of normalized mRNA levels in C1 and manually isolated (MI) cells. (A) Mean values of C1 (single‐cell auto prep system) gene expression (Log2) compared to bulk populations for all 71 genes analyzed in senescent cells. Each point is a single gene. (B) Rank‐order analysis for all 71 genes for either C1 (*y*‐axis) or MI (*x*‐axis) method. (C) Venn diagram showing number of genes significantly changed in senescent cells relative to quiescent cells by C1 or MI methods.

Our initial analysis revealed few significant differences between the two cell populations primarily because senescent cells had a lower overall transcript abundance, possibly owing to less efficient lysis/amplification. After normalization (Livak *et al*., 2013), however, it was possible to identify transcriptional profiles that were consistent with transcriptome analyses of bulk cell populations (Fig. 1A). Normalization also resulted in more coordinated expression profiles between the C1 and MI pools of cells (Fig. 1B; Fig. S3A,B). Following normalization, MI cells showed 61 significantly altered genes during senescence, while C1 showed 31, presumably due to the smaller number of successfully amplified genes. Notably, 28 of the altered genes were shared between C1 and MI cells (Fig. 1C).

**Figure 2:**
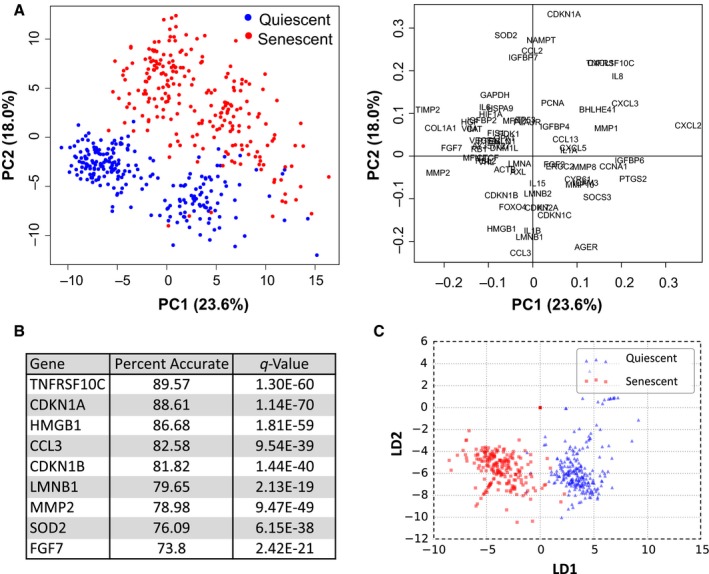
Gene expression signatures that identify senescent cells. (A) Principal component analysis of gene expression data. Plot of all quiescent (blue) and senescent (red) cells using 70 genes (left panel). Variables factor map shows genes used and their contributions to PC1 and PC2 (right panel). (B) Table listing the strongest predictors of senescent cells calculated by linear discriminant analyses (LDA). (C) LDA plot using five genes (CDKN1A, CDKN1B, LMNB1, TNFRS10C, and CCL3) for quiescent (blue) and senescent (red) cells.

Using these datasets, we first asked whether senescent and quiescent cells were distinguishable at the single‐cell level by gene expression signatures. Principal component analysis (PCA) allowed us to identify senescent cells by sorting the first and second principal components (consisting of a combination of expression values for 70 genes) (Fig. 2A, right panel). When plotted in two dimensions (X–Y axes), quiescent and senescent populations clustered independently (Fig. 2A, left panel). Senescent cells displayed a larger spread of values, consistent with more variability.

Using the expression of all 70 genes, linear discriminant analysis (LDA) correctly classified > 98% of cells as senescent or quiescent. Furthermore, expression of several genes individually strongly predicted senescence status. The two most predictive genes were *TNFRSF10C* and *CDNK1A* (Fig. 2B). *TNFRSF10C* encodes a secreted decoy receptor that prevents TRAIL‐induced apoptosis (Sheridan *et al*., 1997). This gene is induced in response to genotoxic stress in a p53‐dependent manner (Sheikh *et al*., 1999) and is a component of the SASP (identified by its alias TRAILR3) (Coppe *et al*., 2008). *CDKN1A*, which encodes p21^Waf1^, is a cyclin‐dependent kinase inhibitor (CDKi) and primary mediator of both the transient and permanent (senescence) p53‐induced G1 arrests (El‐Deiry *et al*., 1993; Dulic *et al*., 1994). CDKN2A, which encodes p16^INK4a^, is another CDKi, and common marker of senescence and aging (Beausejour *et al*., 2003; Krishnamurthy *et al*., 2004), but was not assayed in these analyses owing to primer failures. Reduced *LMNB1* or *HMGB1* expression also strongly predicted senescence. Both gene products are lost from the nuclei of senescent cells in a p53‐dependent manner (Freund *et al*., 2012; Davalos *et al*., 2013). As bleomycin causes genotoxic stress and activates p53, *CDKN1A*,* TNFRSF10C*,* LMNB1,* and *HMGB1* are likely markers of p53 activation during senescence. Indeed, the combination of *CDKN1A*,* CDKN1B*,* LMNB1*,* TNFRSF10C,* and *CCL3* was sufficient to predict senescence in 97% of cells (*P *= 9.54E‐39) by LDA (Fig. 2C). Thus, markers of p53 activity are strong indicators of senescence at the single‐cell level.

A few quiescent cells associated more strongly with senescent cells using both PCA and LDA (Fig 2A,C). As even early passage fibroblast populations often contain a small percentage of senescent cells (Dimri *et al*., 1995), this association was expected. Together, these analyses indicate that senescent cells can be identified at the single‐cell level based upon their gene expression signatures.

### Senescent cells display more variability in mRNA levels than quiescent cells

**Figure 3:**
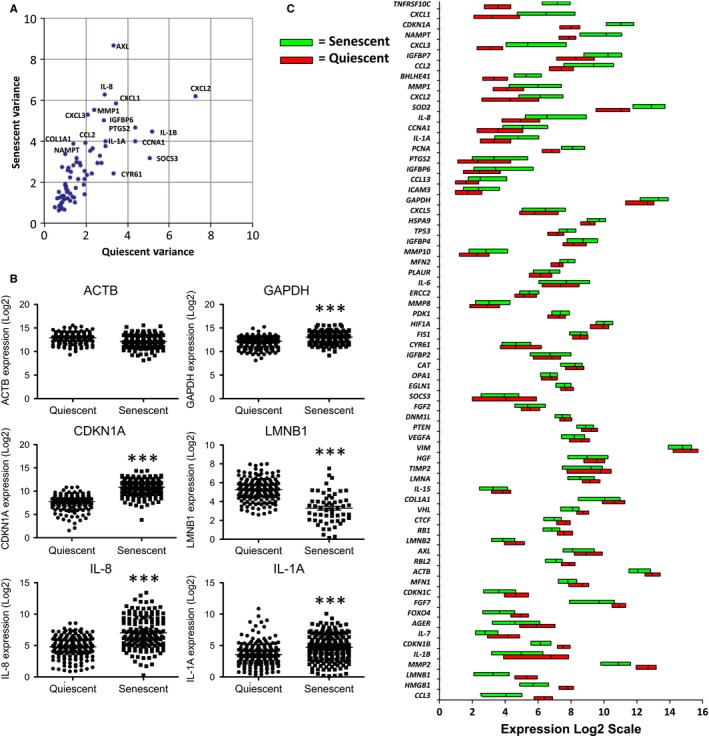
Increased gene expression variability in senescent cells. (A) Plot of coefficients of variation of senescent (*y*‐axis) vs. quiescent (*x*‐axis) cells for each gene analyzed. Noteworthy genes are labeled in the figure. (B) Expression plots of each cell analyzed for control genes (*ACTB* and *GAPDH*), a p53‐inducible senescence gene (*CDKN1A*), a p53‐repressed senescence gene (*LMNB1*), and two SASP genes (*IL‐8* and *CXCL3*). *** = *P *< 0.0001 (C) Box plots showing the first quartile, median, and third quartile of the expression level for each gene analyzed in senescent (green) and quiescent (red) cells.

There is increased variability in selected mRNA levels among single cardiomyocytes from aged, compared to young, mice, and in mouse fibroblasts treated with H_2_O_2_ (Bahar *et al*., 2006). Both age and H_2_O_2_ increase the number of senescent cells. In agreement with these studies, many of the genes (51%) assayed here displayed significantly increased variability in mRNA levels among single cells following induction of senescence. This increased variability was not universal, however, with a few genes (7%) (such as *CYR61* and *SOCS3*) showing lower variance in senescent compared to quiescent cells (Fig. 3A). Control genes, known to show little change in expression in senescent cells, generally displayed only minor changes in variance. For example, *GAPDH* displayed a nonsignificant (*P* > 0.05, Monte Carlo permutation test) decrease in variability, whereas *ACTB* variability increased slightly (Fig. 3A–C). Interestingly, *CDKN1A* also showed no significant increases in variance (*P *> 0.05), despite being induced in senescent cells (Fig. 3B).

Senescence‐associated variability in gene expression could result from genomic rearrangements, as suggested for aged and H_2_O_2_‐treated cells (Bahar *et al*., 2006), but also from changes in transcriptional activation or repression in senescent cells. *LMNB1* mRNA levels, which decline in senescent cells (Freund *et al*., 2012), also showed increased variability (Fig. 3B) accompanied, as expected, by fewer transcripts with detectable amplification. Similarly, many SASP factors, such as *IL‐8* and *IL‐1A*, showed strongly increased variability, with a subset of cells displaying a very high level of gene expression (Fig. 3B). This finding suggests the increase in mRNAs encoding SASP factors (Coppe *et al*., 2008) could result from contributions by only a subset of senescent cells at any time.

### Gene expression correlations change during cellular senescence

**Figure 4:**
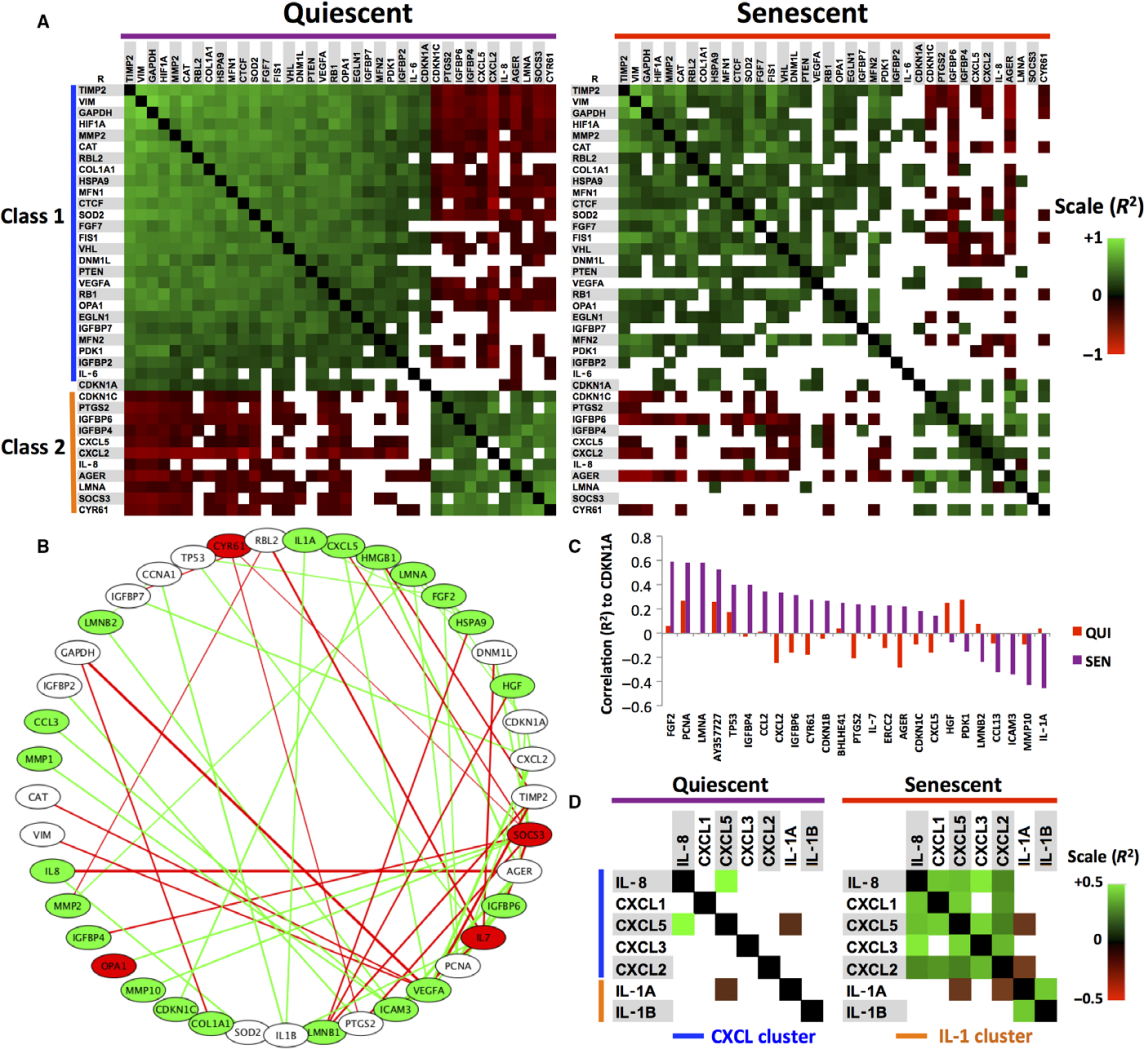
Gene expression correlations in quiescent and senescent cells. (A) Heat maps of Pearson's coefficients (R^2^) between clusters of coexpressed genes in quiescent (left panel) and senescent (right panel) cells. Green indicates positive correlations, red indicates negative correlations, and empty cells indicate nonsignificant (*P *> 0.01) correlations. Two classes of directly (green) and indirectly (red) correlated expression patterns (labeled Class 1 and Class 2) appear across multiple genes. (B) Circular node network indicating changes in correlations between quiescent and senescent cells. Number of altered correlations increases from left to right. Green nodes indicate genes with increased variability in senescent cells, while red nodes indicate decreased variability; white nodes indicate no change. Connecting lines indicate whether correlations increase (green) or decrease (red) between genes. (C) Correlation (R^2^) levels of multiple genes relative to CDKN1A. Violet bars indicate quiescent cells, whereas red bars indicate senescent cells. (D) Heat map of correlations (R^2^) between genes in the CXCL (blue) and IL‐1 (orange) clusters in quiescent (left) and senescent (right) cells, as in A.

Are cells that strongly express SASP factors such as *IL‐8* and *IL‐1A* a subset of senescent cells, or do individual cells express these and other senescence‐associated transcripts in variable quantities? To address these questions, we calculated correlation coefficients (R^2^) for all genes, eliminating nonsignificant (*P* > 0.01) correlations (Fig. S4). Two classes of inversely correlated genes emerged from analyses of quiescent cells (arbitrarily labeled ‘Class 1’ and ‘Class 2’) (Fig. 4A, left). From analyses of senescent cells, these classes were largely retained, but most correlation values declined (Fig. 4A, right), with many no longer meeting the *P*‐value threshold. Pathway analysis of Class 1 genes showed a strong relationship between extracellular matrix regulation, cellular stress responses (including hypoxic responses), and cytokine signaling, whereas Class 2 genes most strongly associated with inflammatory responses (Fig. S5).

Most of the declines in correlation were likely the result of increased variability. However, a subset of genes showed increased correlations (ΔR^2^ > 0.5; R^2^ (sen) > R^2^ (qui)), either directly or inversely (Fig. 4A). Among these genes, there was no relationship between variability in transcript abundance and correlation among paired transcripts (Fig. 4B), suggesting that variability alone cannot account for these increases.

Of all genes that displayed highly altered correlations with senescence, *CDKN1A* (which was consistently induced in senescent cells; Fig. 3B) was most notable, displaying increased correlations with 25 gene transcripts (Fig. 4C). Furthermore, *CDKN1A* showed a substantial shift in its correlation patterns, losing correlation with some genes (Class 1) and gaining correlation with others (Class 2) (Fig. 4A).

As many SASP factors are strongly clustered in the genome (Coppe *et al*., 2010), we asked whether genomic location is linked to expression coregulation. Supporting this possibility, transcripts from the IL‐1 cluster (*IL‐1A* and *IL‐1B*, promoters separated by ~50 kb), as well as the CXCL cluster (in order: *IL‐8, CXCL1, CXCL5, CXCL3,* and *CXCL2,* separated in total by ~360 kb), went from nonsignificant correlations to stronger, significant direct correlations, suggesting these genes were induced in a coordinated manner (Fig. 4D). By comparison, little to no correlation of expression was observed when the IL‐1 cluster was analyzed against the CXCL cluster (Fig. 4D), which are on different chromosomes. These data suggest that genomic organization can influence gene expression changes in single cells.

Together, our correlation data indicate that, whereas the expression of many genes is coordinated under quiescent conditions, some senescence‐specific gene expression processes appear to be regulated independently of each other.

## Discussion

As senescent cells are relatively rare, even in tissues from aged animals (Dimri *et al*., 1995; Herbig *et al*., 2006; Kreiling *et al*., 2011; Waaijer *et al*., 2012; Baker *et al*., 2016), the ability to identify and study individual senescent cells is a unique opportunity to better understand the range of senescent phenotypes that contribute to their physiological and pathological effects (Munoz‐Espin & Serrano, 2014). In this study, we highlight the ability to identify senescent cells by their gene expression profile, while also demonstrating that the gene expression profiles of senescent cells are highly heterogeneous. Despite this heterogeneity, genes that reside in closely linked loci appear to be coregulated during senescence.

Identifying senescent cells at the single‐cell level is an important technological step for future studies, especially in human tissues. While transgenic mouse models now allow senescent cells to be identified and isolated from mouse tissues (Demaria *et al*., 2014), identifying senescent cells in human tissues remains difficult. Our study provides proof of principle that single‐cell analyses have potential for identifying senescent cells from human tissues.

Our findings emphasize the risk of using a single biomarker to identify senescent cells, whether in culture or *in vivo*. We recommend using several makers—in our own studies, for example, we tend to use combinations of SA‐Bgal activity, loss of LMNB1 expression, HMGB1 relocalization, p16^INK4a^ and/or p21^WAF1^ expressions, and the expression of strongly upregulated SASP factors. As many inducers of senescence (e.g., telomere attrition, ionizing radiation, bleomycin, and oncogene activation) ultimately induce a DNA damage response, it is likely that many of the factors identified in this study are common to several senescence inducers. However, inducers of senescence that do not cause genotoxic stress (e.g., mitochondrial dysfunction‐associated senescence) (Wiley *et al*., 2016) can have a distinct SASP and gene expression profile and so would need to be identified by those signatures.

Increased gene expression variability in senescent cells is consistent with studies showing such variability during aging and in response to oxidative stress (Bahar *et al*., 2006). Our findings show that senescence‐associated mRNA levels can vary from cell to cell. For example, increases in *CDKN1A* mRNA were tightly clustered, possibly reflecting uniform p53 activation following genotoxic stress (bleomycin administration). By comparison, *LMNB1* and many SASP factors, showing decreased or increased expression, respectively, displayed large variability in expression levels in senescent cells. These data suggest the mechanisms governing the expression of these genes are subject to more stochastic events than those that govern *CDKN1A* expression. Alternatively, genes that show large expression variability might fluctuate temporally, which, in an asynchronous population, would result in cell‐to‐cell differences in the expression levels at any given time.

The increased correlation between genes clustered within genomic loci suggests a level of gene regulation that has not previously been described for senescent cells. One possibility is that senescence‐associated epigenetic changes extend over selected loci, as opposed to individual genes, thereby affecting the accessibility of transcription factors to linked genes within those loci. Indeed, the high mobility group box proteins, which bind non‐B‐type DNA, have been linked to both senescence and the SASP. HMGB1 is lost from the nuclei of senescent cells (Davalos *et al*., 2013), whereas HMGB2 localizes to the promoters of several SASP genes (Aird *et al*., 2016). This altered chromatin landscape may explain the coordinated expression of SASP genes that lie in close genomic proximity. Alternatively, as the correlated genes are regulated by similar transcription factors (such as NF‐κB and C/EBPβ) and likely emerged as a result of genomic duplication, it is possible that their close physical proximity allows transcription factors that leave one gene promoter for other promoters in close proximity.

An important limitation to this and similar single‐cell transcription‐based studies is that mRNA transcript levels may not reflect the steady‐state levels of protein. Furthermore, as noted above, single‐cell analyses currently score transcript levels at a single time point. Nonetheless, our analyses indicate that, at any given time, there are both uniformity in patterns and variability in individual mRNA levels in senescent cell populations.

## Experimental procedures

### Cell culture

IMR‐90 human fetal lung fibroblasts at population doubling level 25–30 were cultured in Dulbecco's modified Eagle's medium (DMEM) supplemented with 10% fetal bovine serum (FBS) and penicillin/streptomycin (growth medium). Cells were seeded at 1 x 10^4^ cells/cm^2^ in 175‐cm^2^ flasks, maintained at 37° C, 10% CO_2,_ and 3% O_2_, and were mycoplasma negative. For senescence induction, cells were given growth medium containing 50 μm bleomycin (senescent) or vehicle (PBS—nonsenescent control) in atmospheric oxygen for 3 hrs. The cells were then washed, given growth medium, and returned to 3% O_2_. The growth medium was changed every 3 days. On day 7, it was replaced with low serum (0.2% FBS) medium, which induced quiescence in control cells. Cells were harvested by trypsinization for analysis on Day 10 (see Fig. S1A). A subset of cells was plated for senescence assays (below).

### Senescence‐associated beta‐galactosidase (SA‐Bgal) assay

Ten days after bleomycin or control treatment, a 12‐well plate was seeded with three wells from each treatment (30 000 cells/well) and allowed to attach overnight. The assay was performed as described (Dimri *et al*., 1995) using a commercial kit (BioVision, Milpitas, CA, USA).

### Measurement of IL‐6 secretion

Quiescent or senescent cells were seeded as for the SA‐Bgal assay and allowed to attach overnight. The following day, the medium was replaced with 500 μL of serum‐free DMEM. Conditioned media were collected 24 hrs later. An ELISA kit (AlphaLISA, Perkin Elmer Biosciences, San Jose, CA, USA) was used to measure IL‐6 in conditioned media according to the manufacturer's instructions; IL‐6 levels were normalized to cell number.

### EdU labeling

Quiescent or senescent cells were plated into four‐well chamber slides (Labtech, Tampa, FL, USA) and allowed to attach overnight. The next day, the cells were given low serum medium containing 10 μm EdU (Invitrogen, Carlsbad, CA, USA). After 24 hrs, cells were washed with PBS, fixed in 4% formalin, then permeabilized in 0.5% Triton X‐100 for 30 min, and washed twice in ice‐cold PBS. Permeabilized cells were subjected to the EdU reaction according to the manufacturer's instructions (Click‐IT EdU Imaging Kit, Invitrogen).

### Manual isolation of single cells

Cells cultured as described above were trypsinized, pelleted by centrifugation, and resuspended in Krebs–Henseleit buffer. Approximately 20 000 cells were placed in 10 mL of buffer in a 15‐cm dish. Under phase‐contrast microscopy, a small glass needle was used to mouth pipette individual cells into 200‐μL reaction tubes in less than 2 μL volumes. Single cells were immediately frozen on dry ice and maintained at −80° C until analysis.

### Isolation of single cells by C1

Single cells were captured in the C1 platform (Fluidigm Inc., South San Francisco, CA, USA) as per the manufacturer's instructions (Fluidigm) and verified as single‐cell captures by microscopic analysis of each nanofluidic chamber. Chambers on the C1 chip containing more than one cell were excluded from subsequent analysis.

### Measurement of single‐cell transcriptional profiles

Gene expression in single cells was measured by quantitative multiplex PCR following pre‐amplification using the Biomark platform (Fluidigm) according to the manufacturer's instructions. Data were normalized as described (Livak *et al*., 2013). Briefly, the data adjustment was as follows: Log2 expression data were extracted through the single‐cell analysis package for all PCR plates, including both C1 and manual isolation data. The data were then categorized as deriving from quiescent or senescent cells prior to subsequent analyses.

### Principle component analysis (PCA)

PCA was performed on qPCR values of 70 genes from 464 individual cells, 235 quiescent cells, and 229 senescent cells (one senescent cell was removed because none of its transcript was detected by qPCR) using the pcaMethods package in R (probability principle component analysis method with missing values imputed by default).

### Linear discriminant analysis (LDA)

LDA was performed to maximize the separation between the 235 quiescent and 230 senescent cells. To determine which gene subsets were most important in correctly classifying cells as quiescent or senescent, we used a sequential backward selection process (Cotter *et al*., 2001). Starting with all the data, we used LDA to obtain the maximum separation between quiescent and senescent cells. Then, the loss of a single transcription vector was tested by removing each gene and repeating the LDA analysis with the data from the remaining 70 genes. The gene loss having the smallest impact on the ability to separate quiescent from senescent cells was deleted. This process was repeated until only the most predictive genes for discrimination remained. Because imputation of missing expression values can influence the results of LDA and the backward selection process, three separate imputation schemes were employed, with the final ranking taking each of these gene rankings into account. Missing values were first set to zero, then to the average of all values recorded for that gene, and finally to the average of all quiescent or senescent values.

### Pathway analysis

Class 1 and Class 2 gene lists were submitted to the Database for Annotation, Visualization and Integrated Discovery (DAVID v.6.7) (Huang *et al*., 2007) for annotation enrichment analysis. The full human genome was used as a background. Pathways and annotations showing significant enrichment following Benjamini multiple testing correction are summarized in Fig. S4:

## Funding

American Federation for Aging Research, (Grant / Award Number: ‘Fellowship’) National Institute on Aging, (Grant / Award Number: ‘AG009909‘,’AG017242‘,’T32‐AG000266‘) National Institute of Arthritis and Musculoskeletal and Skin Diseases, (Grant / Award Number: ‘AR063919‘)

## Conflict of interest

RL, JS, and MU were employed by the Fluidigm Corporation (South San Francisco) at the time C1 analyses were performed, and C1 analyses were performed at Fluidigm.

## Author contributions

CW and JF designed the experiments, manually isolated single cells, and analyzed the data under the guidance of JC and SM. CW selected the genes that were analyzed, prepared senescent and quiescent cells, and performed several analyses. JF ran all qPCRs, normalized the data, and performed several analyses. CM performed LDA. XD conducted PCA with guidance from JV. RL and JS performed C1‐based isolation of cells and mRNA under the guidance of MU. CW and JC wrote the article with input from SM and JV.

## Supporting information


**Fig. S1** Experimental design and quality control. (A) Schematic representation of the experimental design showing time points for generating the datasets and assays to confirm quiescence and senescence. Purple indicates quiescent cells treated with vehicle (PBS), whereas red indicates senescent cells treated with bleomycin. (B–C) Quality control assays confirming the induction of senescence. (B) Senescence‐associated beta galactosidase (SA‐B‐gal) and EdU incorporation analysis of quiescent (PBS‐treated) and senescent (bleomycin‐treated) samples. (C) IL‐6 ELISA confirming establishment of a SASP in senescent cells.
**Fig. S2** Detection of gene transcripts varies by method and cell type. (A) Rank ordered success rates by gene for all samples in our data sets. (B) Success rates by gene for C1 vs. MI methods. (C) Success rates by gene for either senescent (SEN) or quiescent (QUI) cell populations.
**Fig. S3** Normalization of datasets. Density maps for gene expression analyzed by C1 or manual isolation (MI) before (A) and after (B) normalization.
**Fig. S4** Examples of genes with strong positive or negative correlations. Relative gene expression values for each single cell were plotted against each other. Each axis indicates gene expression values for each single cell; red dots indicate senescent cells, blue dots indicate quiescent cells. (A) Example of a strong positive correlation: GAPDH plotted against vimentin. (B) Example of a strong negative correlation: AGER plotted against GAPDH.
**Fig. S5** Pathway analysis of Class 1 and Class 2 genes. Pathway enrichment analysis of Class 1 (above) and Class 2 (below) genes, sorted by *P*‐value.

**Data S1** Single cell expression datasheet.

## References

[acel12632-bib-0001] Aird KM , Iwasaki O , Kossenkov AV , Tanizawa H , Fatkhutdinov N , Bitler BG , Le L , Alicea G , Yang TL , Johnson FB , Noma KI , Zhang R (2016) HMGB2 orchestrates the chromatin landscape of senescence‐associated secretory phenotype gene loci. J. Cell Biol. 215, 325–334. 27799366 10.1083/jcb.201608026 PMC5100296

[acel12632-bib-0002] Bahar R , Hartmann CH , Rodriguez KA , Denny AD , Busuttil RA , Dolle ME , Calder RB , Chisholm GB , Pollock BH , Klein CA , Vijg J (2006) Increased cell‐to‐cell variation in gene expression in ageing mouse heart. Nature 441, 1011–1014. 16791200 10.1038/nature04844

[acel12632-bib-0003] Baker DJ , Childs BG , Durik M , Wijers ME , Sieben CJ , Zhong J , Saltness RA , Jeganathan KB , Verzosa GC , Pezeshki A , Khazaie K , Miller JD , van Deursen JM (2016) Naturally occurring p16(Ink4a)‐positive cells shorten healthy lifespan. Nature 530, 184–189. 26840489 10.1038/nature16932 PMC4845101

[acel12632-bib-0004] Beausejour CM , Krtolica A , Galimi F , Narita M , Lowe SW , Yaswen P , Campisi J (2003) Reversal of human cellular senescence: roles of the p53 and p16 pathways. EMBO J. 22, 4212–4222. 12912919 10.1093/emboj/cdg417 PMC175806

[acel12632-bib-0005] Campisi J (2013) Aging, cellular senescence, and cancer. Annu. Rev. Physiol. 75, 685–705. 23140366 10.1146/annurev-physiol-030212-183653 PMC4166529

[acel12632-bib-0006] Coppe JP , Patil CK , Rodier F , Sun Y , Munoz DP , Goldstein J , Nelson PS , Desprez PY , Campisi J (2008) Senescence‐associated secretory phenotypes reveal cell‐nonautonomous functions of oncogenic RAS and the p53 tumor suppressor. PLoS Biol. 6, 2853–2868. 19053174 10.1371/journal.pbio.0060301 PMC2592359

[acel12632-bib-0007] Coppe JP , Patil CK , Rodier F , Krtolica A , Beausejour CM , Parrinello S , Hodgson JG , Chin K , Desprez PY , Campisi J (2010) A human‐like senescence‐associated secretory phenotype is conserved in mouse cells dependent on physiological oxygen. PLoS ONE 5, e9188. 20169192 10.1371/journal.pone.0009188 PMC2820538

[acel12632-bib-0008] Cotter SF , Kreutz‐Delgado K , Rao BD (2001) Backward sequential elimination for sparse vector subset selection. Signal Process. 81, 1849–1864.

[acel12632-bib-0009] Davalos AR , Kawahara M , Malhotra GK , Schaum N , Huang J , Ved U , Beausejour CM , Coppe JP , Rodier F , Campisi J (2013) p53‐dependent release of Alarmin HMGB1 is a central mediator of senescent phenotypes. J. Cell Biol. 201, 613–629. 23649808 10.1083/jcb.201206006 PMC3653366

[acel12632-bib-0010] Demaria M , Ohtani N , Youssef SA , Rodier F , Toussaint W , Mitchell JR , Laberge RM , Vijg J , Van Steeg H , Dolle ME , Hoeijmakers JH , de Bruin A , Hara E , Campisi J (2014) An essential role for senescent cells in optimal wound healing through secretion of PDGF‐AA. Dev. Cell 31, 722–733. 25499914 10.1016/j.devcel.2014.11.012 PMC4349629

[acel12632-bib-0011] Dimri GP , Lee X , Basile G , Acosta M , Scott G , Roskelley C , Medrano EE , Linskens M , Rubelj I , Pereira‐Smith OM , Peacocke M , Campisi J (1995) A novel biomarker identifies senescent human cells in culture and in aging skin in vivo. Proc. Natl Acad. Sci. USA 92, 9363–9367. 7568133 10.1073/pnas.92.20.9363 PMC40985

[acel12632-bib-0012] Dulic V , Kaufmann WK , Wilson SJ , Tlsty TD , Lees E , Harper JW , Elledge SJ , Reed SI (1994) p53‐dependent inhibition of cyclin‐dependent kinase activities in human fibroblasts during radiation‐induced G1 arrest. Cell 76, 1013–1023. 8137420 10.1016/0092-8674(94)90379-4

[acel12632-bib-0013] El‐Deiry WS , Tokino T , Velculescu VE , Levy DB , Parsons R , Trent JM , Lin D , Mercer WE , Kinzler KW , Vogelstein B (1993) WAF1, a potential mediator of p53 tumor suppression. Cell 75, 817–825. 8242752 10.1016/0092-8674(93)90500-p

[acel12632-bib-0014] Freund A , Laberge RM , Demaria M , Campisi J (2012) Lamin B1 loss is a senescence‐associated biomarker. Mol. Biol. Cell 23, 2066–2075. 22496421 10.1091/mbc.E11-10-0884 PMC3364172

[acel12632-bib-0015] Herbig U , Ferreira M , Condel L , Carey D , Sedivy JM (2006) Cellular senescence in aging primates. Science 311, 1257. 16456035 10.1126/science.1122446

[acel12632-bib-0016] Huang DW , Sherman BT , Tan Q , Kir J , Liu D , Bryant D , Guo Y , Stephens R , Baseler MW , Lane HC , Lempicki RA (2007) DAVID bioinformatics resources: expanded annotation database and novel algorithms to better extract biology from large gene lists. Nucleic Acids Res. 35, W169–W175. 17576678 10.1093/nar/gkm415 PMC1933169

[acel12632-bib-0017] Kreiling JA , Tamamori‐Adachi M , Sexton AN , Jeyapalan JC , Munoz‐Najar U , Peterson AL , Manivannan J , Rogers ES , Pchelintsev NA , Adams PD , Sedivy JM (2011) Age‐associated increase in heterochromatic marks in murine and primate tissues. Aging Cell 10, 292–304. 21176091 10.1111/j.1474-9726.2010.00666.x PMC3079313

[acel12632-bib-0018] Krishnamurthy J , Torrice C , Ramsey MR , Kovalev GI , Al‐Regaiey K , Su L , Sharpless NE (2004) Ink4a/Arf expression is a biomarker of aging. J Clin Invest. 114, 1299–1307. 15520862 10.1172/JCI22475 PMC524230

[acel12632-bib-0019] Livak KJ , Wills QF , Tipping AJ , Datta K , Mittal R , Goldson AJ , Sexton DW , Holmes CC (2013) Methods for qPCR gene expression profiling applied to 1440 lymphoblastoid single cells. Methods 59, 71–79. 23079396 10.1016/j.ymeth.2012.10.004 PMC3562442

[acel12632-bib-0020] Munoz‐Espin D , Serrano M (2014) Cellular senescence: from physiology to pathology. Nat. Rev. Mol. Cell Biol. 15, 482–496. 24954210 10.1038/nrm3823

[acel12632-bib-0021] Orjalo AV , Bhaumik D , Gengler BK , Scott GK , Campisi J (2009) Cell surface‐bound IL‐1alpha is an upstream regulator of the senescence‐associated IL‐6/IL‐8 cytokine network. Proc. Natl. Acad. Sci. U.S.A. 106, 17031–17036. 19805069 10.1073/pnas.0905299106 PMC2761322

[acel12632-bib-0022] Sheikh MS , Huang Y , Fernandez‐Salas EA , El‐Deiry WS , Friess H , Amundson S , Yin J , Meltzer SJ , Holbrook NJ , Fornace AJ Jr (1999) The antiapoptotic decoy receptor TRID/TRAIL‐R3 is a p53‐regulated DNA damage‐inducible gene that is overexpressed in primary tumors of the gastrointestinal tract. Oncogene 18, 4153–4159. 10435597 10.1038/sj.onc.1202763

[acel12632-bib-0023] Sheridan JP , Marsters SA , Pitti RM , Gurney A , Skubatch M , Baldwin D , Ramakrishnan L , Gray CL , Baker K , Wood WI , Goddard AD , Godowski P , Ashkenazi A (1997) Control of TRAIL‐induced apoptosis by a family of signaling and decoy receptors. Science 277, 818–821. 9242611 10.1126/science.277.5327.818

[acel12632-bib-0024] Waaijer ME , Parish WE , Strongitharm BH , van Heemst D , Slagboom PE , de Craen AJ , Sedivy JM , Westendorp RG , Gunn DA , Maier AB (2012) The number of p16INK4a positive cells in human skin reflects biological age. Aging Cell 11, 722–725. 22612594 10.1111/j.1474-9726.2012.00837.x PMC3539756

[acel12632-bib-0025] Wiley CD , Velarde MC , Lecot P , Liu S , Sarnoski EA , Freund A , Shirakawa K , Lim HW , Davis SS , Ramanathan A , Gerencser AA , Verdin E , Campisi J (2016) Mitochondrial dysfunction induces senescence with a distinct secretory phenotype. Cell Metab. 23, 303–314. 26686024 10.1016/j.cmet.2015.11.011 PMC4749409

