# Supplementary material for: Analysis of individual cells identifies cell‐to‐cell variability following induction of cellular senescence
Source: Aging Cell. 2017 Jul 11;16(5):1043–50. doi: 10.1111/acel.12632 (PMC5595671; doi:10.1111/acel.12632)
Supplement: Supplementary file 1 — Fig. S1 Experimental design and quality control. (A) Schematic representation of the experimental design showing time points for generating the datasets and assays to confirm quiescence and senescence. Purple indicates quiescent cells treated with vehicle (PBS), whereas red indicates senescent cells treated with bleomycin. (B–C) Quality control assays confirming the induction of senescence. (B) Senescence‐associated beta galactosidase (SA‐B‐gal) and EdU incorporation analysis of quiescent (PBS‐treated) and senescent (bleomycin‐treated) samples. (C) IL‐6 ELISA confirming establishment of a SASP in senescent cells. Fig. S2 Detection of gene transcripts varies by method and cell type. (A) Rank ordered success rates by gene for all samples in our data sets. (B) Success rates by gene for C1 vs. MI methods. (C) Success rates by gene for either senescent (SEN) or quiescent (QUI) cell populations. Fig. S3 Normalization of datasets. Density maps for gene expression analyzed by C1 or manual isolation (MI) before (A) and after (B) normalization. Fig. S4 Examples of genes with strong positive or negative correlations. Relative gene expression values for each single cell were plotted against each other. Each axis indicates gene expression values for each single cell; red dots indicate senescent cells, blue dots indicate quiescent cells. (A) Example of a strong positive correlation: GAPDH plotted against vimentin. (B) Example of a strong negative correlation: AGER plotted against GAPDH. Fig. S5 Pathway analysis of Class 1 and Class 2 genes. Pathway enrichment analysis of Class 1 (above) and Class 2 (below) genes, sorted by P‐value. [file ACEL-16-1043-s001.pdf]

SUPPORTING INFORMATION

Figure S1. Experimental design and quality control. (A) Schematic representation of the experimental design showing time points for generating the datasets and assays to confirm quiescence and senescence. Purple indicates quiescent cells treated with vehicle (PBS), whereas red indicates senescent cells treated with bleomycin. (B-C) Quality control assays confirming the induction of senescence. (B) Senescence-associated beta galactosidase (SA-B-gal) and EdU incorporation analysis of quiescent (PBS-treated) and senescent (bleomycin-treated) samples. (C) IL-6 ELISA confirming establishment of a SASP in senescent cells.

Figure S2. Detection of gene transcripts varies by method and cell type. (A) Rank ordered success rates by gene for all samples in our data sets. (B) Success rates by gene for C1 vs MI methods. (C) Success rates by gene for either senescent (SEN) or quiescent (QUI) cell populations.

Figure S3. Normalization of datasets. Density maps for gene expression analyzed by C1 or MI before (A) and after (B) normalization.

Figure S4. Examples of genes with strong positive or negative correlations. Relative gene expression values for each single cell were plotted against each other. Each axis indicates gene expression values for each single cell; red dots indicate senescent cells, blue dots indicate quiescent cells. (A) Example of a strong positive correlation: GAPDH plotted against vimentin. (B) Example of a strong negative correlation: AGER plotted against GAPDH.

Figure S5. Pathway analysis of Class 1 and Class 2 genes. Pathway enrichment analysis of Class 1 (above) and Class 2 (below) genes, sorted by p-value.

Figure S1

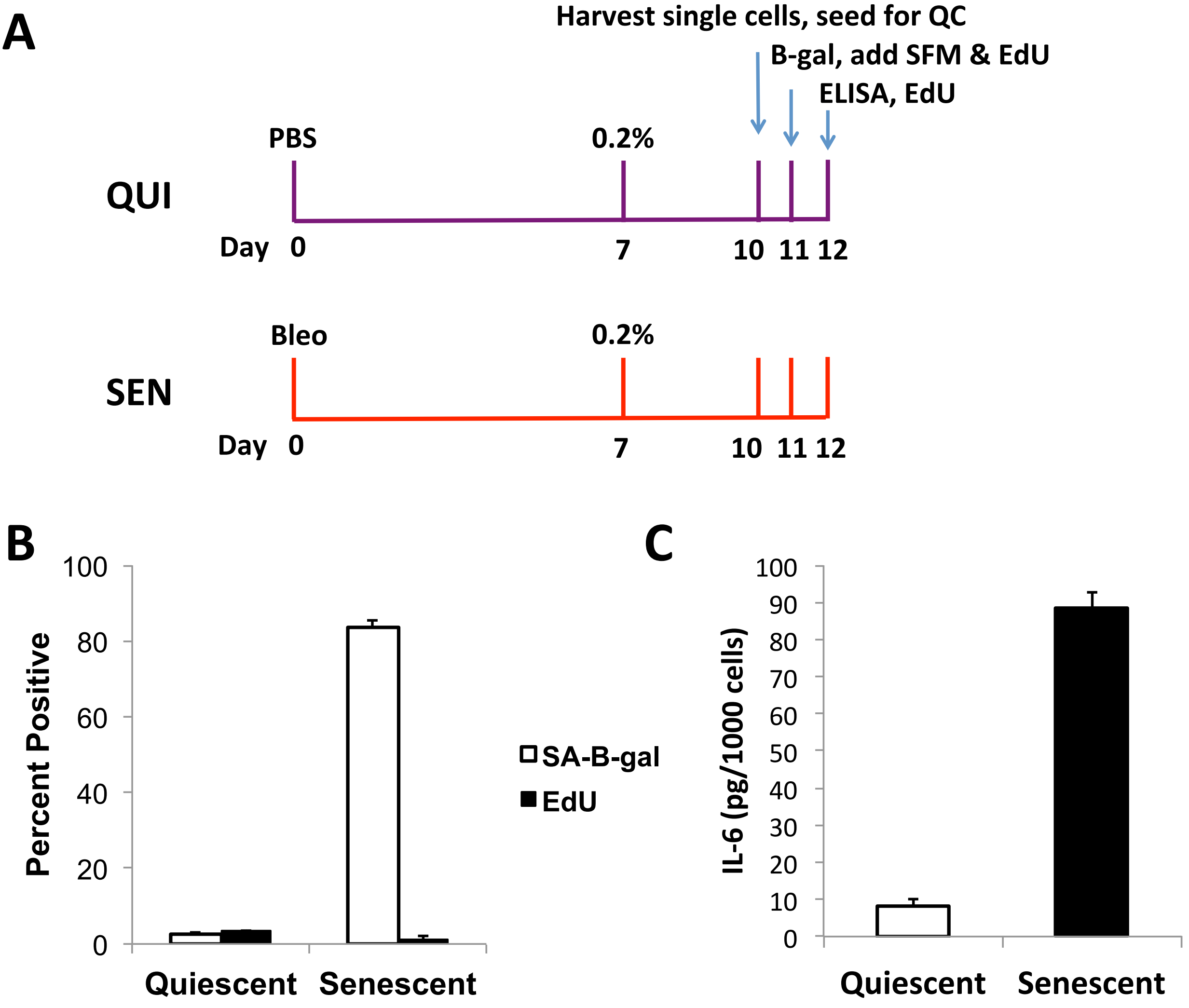

A
Harvest single cells, seed for QC
B-gal, add SFM & EdU
ELISA, EdU
PBS
0.2%
QUI
Day 0
7
10 11 12
Bleo
0.2%
SEN
Day 0
7
10 11 12
B
Percent Positive
100
80
60
40
20
0
Quiescent
Senescent
SA-B-gal
EdU
C
IL-6 (pg/1000 cells)
100
90
80
70
60
50
40
30
20
10
0
Quiescent
Senescent

Figure S2

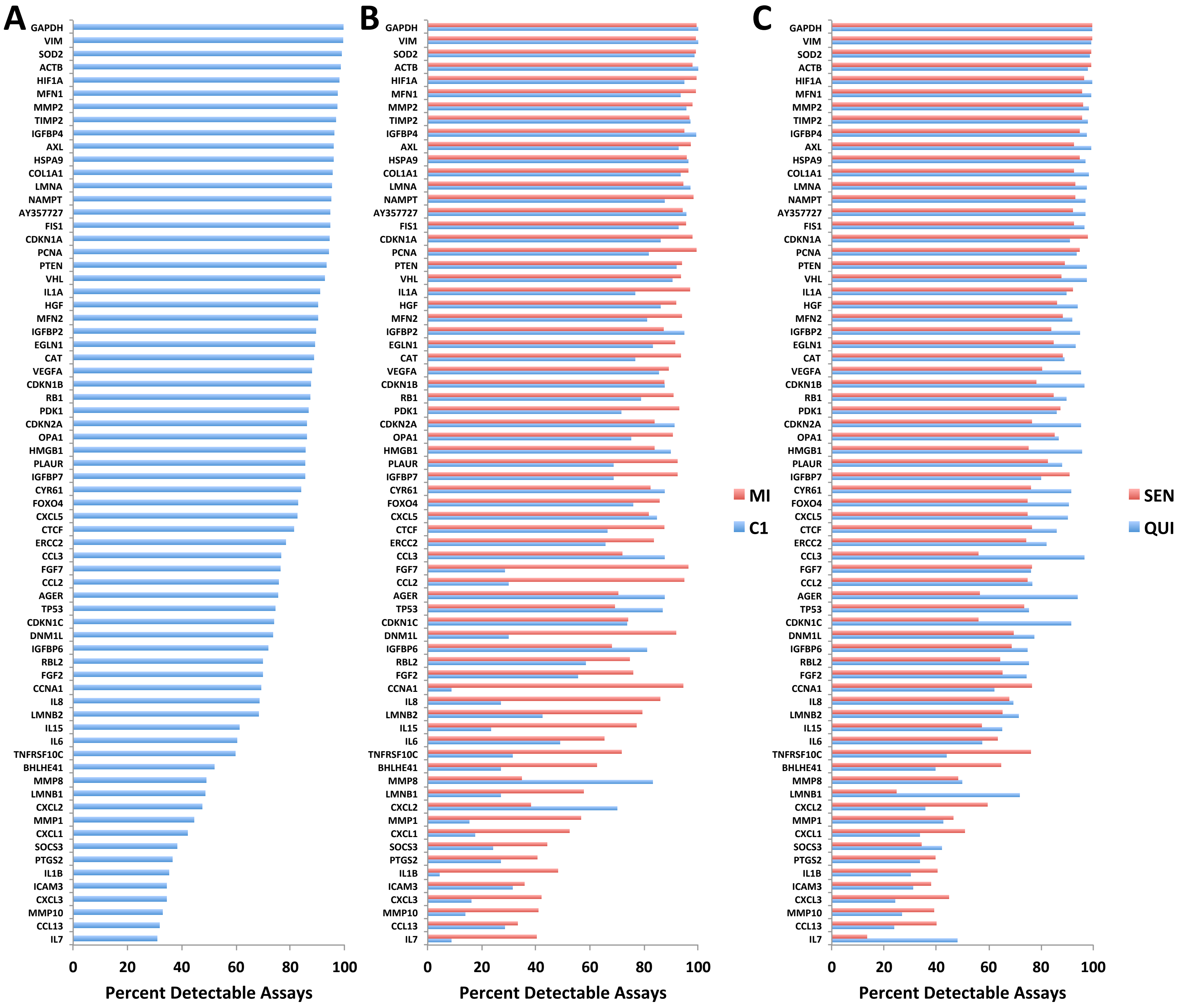

A
B
C
GAPDH
VIM
SOD2
ACTB
HIF1A
MFN1
MMP2
TIMP2
IGFBP4
AXL
HSPA9
COL1A1
LMNA
NAMPT
AY357727
FIS1
CDKN1A
PCNA
PTEN
VHL
IL1A
HGF
MFN2
IGFBP2
EGLN1
CAT
VEGFA
CDKN1B
RB1
PDK1
CDKN2A
OPA1
HMGB1
PLAUR
IGFBP7
CYR61
FOXO4
CXCL5
CTCF
ERCC2
CCL3
FGF7
CCL2
AGER
TP53
CDKN1C
DNM1L
IGFBP6
RBL2
FGF2
CCNA1
IL8
LMNB2
IL15
IL6
TNFRSF10C
BHLHE41
MMP8
LMNB1
CXCL2
MMP1
CXCL1
SOCS3
PTGS2
IL1B
ICAM3
CXCL3
MMP10
CCL13
IL7
0
20
40
60
80
100
Percent Detectable Assays
MI
C1
SEN
QUI

Figure S3

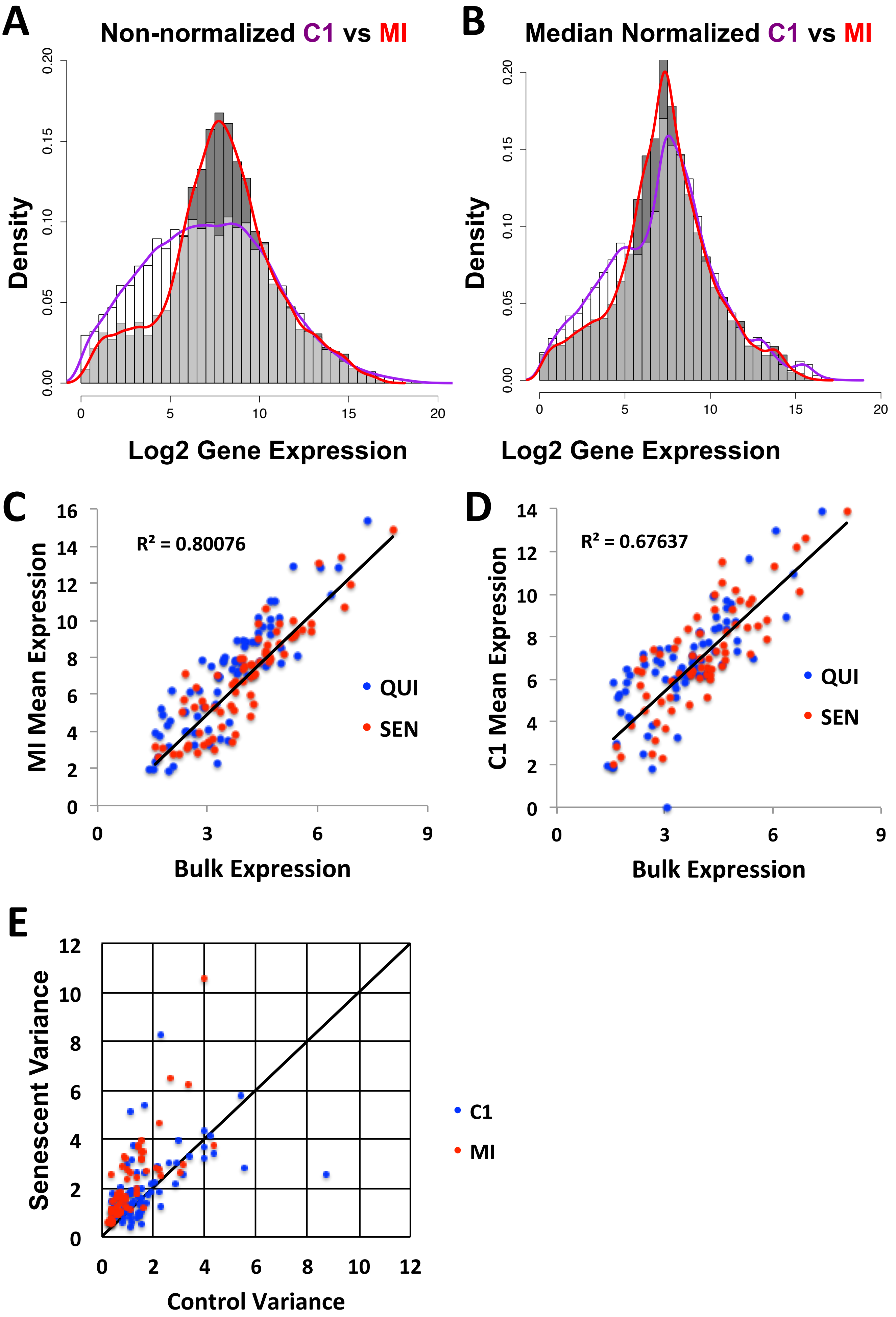

A
Non-normalized C1 vs MI
Density
Log2 Gene Expression
B
Median Normalized C1 vs MI
Density
Log2 Gene Expression
C
MI Mean Expression
R^2 = 0.80076
QUI
SEN
Bulk Expression
D
C1 Mean Expression
R^2 = 0.67637
QUI
SEN
Bulk Expression
E
Senescent Variance
C1
MI
Control Variance

Figure S4

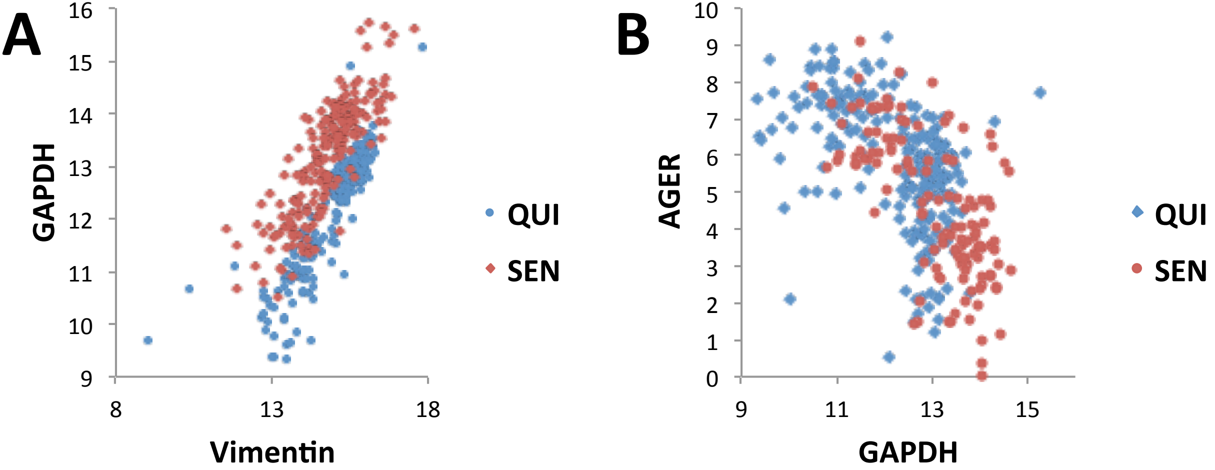
A
16
15
14
13
12
11
10
9
GAPDH
8
13
18
Vimentin
QUI
SEN
B
10
9
8
7
6
5
4
3
2
1
0
AGER
9
11
13
15
GAPDH
QUI
SEN

Figure S5

Class 1 pathways

Pathway name	Entities pValue	Entities FDR	Submitted entities found
Activation of Matrix Metalloproteinases	1.11E-16	1.48E-14	MMP2;TIMP2
Degradation of the extracellular matrix	1.11E-16	1.48E-14	COL1A1;MMP2;TIMP2
Collagen degradation	1.11E-16	1.48E-14	COL1A1;MMP2
Extracellular matrix organization	1.11E-13	1.11E-11	COL1A1;MMP2;TIMP2
Interleukin-4 and 13 signaling	3.64E-11	2.91E-09	IL6;MMP2;VIM;HIF1A;VEGFA
Cellular responses to stress	7.07E-09	4.66E-07	EGLN1;HSPA9;RB1;IL6;CAT;IGFBP7;VHL;SOD2;HIF1A;VEGFA
Cellular responses to external stimuli	5.12E-08	2.92E-06	EGLN1;HSPA9;RB1;IL6;CAT;IGFBP7;VHL;SOD2;HIF1A;VEGFA
Signaling by Interleukins	3.20E-07	1.60E-05	FGF7;IL6;MMP2;VIM;VHL;HIF1A;VEGFA
Immune System	5.19E-06	2.18E-04	COL1A1;IL6;FGF7;MMP2;CAT;TIMP2;PTEN;VHL;VIM;HIF1A;PDK1;VEGFA
Cytokine Signaling in Immune system	5.46E-06	2.18E-04	FGF7;IL6;MMP2;VIM;VHL;HIF1A;VEGFA
Assembly of collagen fibrils and other multimeric structures	7.38E-06	2.66E-04	COL1A1;MMP2
Regulation of Hypoxia-inducible Factor (HIF) by oxygen	2.17E-05	6.52E-04	EGLN1;VHL;HIF1A;VEGFA
Cellular response to hypoxia	2.17E-05	6.52E-04	EGLN1;VHL;HIF1A;VEGFA
Regulation of gene expression by Hypoxia-inducible Factor	3.83E-05	1.07E-03	HIF1A;VEGFA
Collagen formation	5.96E-05	1.55E-03	COL1A1;MMP2
Cellular Senescence	1.36E-04	3.41E-03	RB1;IL6;IGFBP7
Regulation of Insulin-like Growth Factor (IGF) transport and uptake	1.51E-04	3.47E-03	IL6;MMP2;IGFBP2;IGFBP7
PIP3 activates AKT signaling	3.15E-04	4.82E-03	FGF7;PTEN;PDK1
PI3K/AKT activation	3.44E-04	4.82E-03	FGF7;PTEN;PDK1
GAB1 signalosome	3.44E-04	4.82E-03	FGF7;PTEN;PDK1
Senescence-Associated Secretory Phenotype (SASP)	4.41E-04	6.18E-03	IL6;IGFBP7
Downstream signaling events of B Cell Receptor (BCR)	1.97E-03	2.56E-02	FGF7;PTEN;PDK1
Oxygen-dependent proline hydroxylation of HIF1-Alpha	2.76E-03	3.41E-02	EGLN1;VHL;HIF1A
Role of LAT2/NTAL/LAB on calcium mobilization	2.84E-03	3.41E-02	FGF7;PTEN;PDK1
Pink/Parkin Mediated Mitophagy	3.52E-03	4.23E-02	MFN1;MFN2

Class 2 pathways

Pathway name	Entities pValue	Entities FDR	Submitted entities found
Chemokine receptors bind chemokines	1.15E-08	1.38E-06	IL8;CXCL2;CXCL5
Interleukin-10 signaling	2.29E-07	1.37E-05	IL8;PTGS2;CXCL2
Peptide ligand-binding receptors	1.07E-05	4.29E-04	IL8;CXCL2;CXCL5
Interleukin-4 and 13 signaling	1.64E-05	4.92E-04	SOCS3;IL8;PTGS2
Signaling by Interleukins	5.81E-05	1.16E-03	SOCS3;IL8;PTGS2;CXCL2
Unfolded Protein Response (UPR)	1.04E-04	1.77E-03	IL8;LMNA
G alpha (i) signalling events	1.31E-04	1.97E-03	IL8;CXCL2;CXCL5
Class A/1 (Rhodopsin-like receptors)	3.28E-04	3.28E-03	IL8;CXCL2;CXCL5
Cytokine Signaling in Immune system	6.12E-04	4.90E-03	SOCS3;IL8;PTGS2;CXCL2
Regulation of Insulin-like Growth Factor (IGF) transport and uptake	1.00E-03	8.00E-03	IGFBP4;IGFBP6;CYR61
